# Diabetes Mellitus and Ischemic Heart Disease: The Role of Ion Channels

**DOI:** 10.3390/ijms19030802

**Published:** 2018-03-10

**Authors:** Paolo Severino, Andrea D’Amato, Lucrezia Netti, Mariateresa Pucci, Marialaura De Marchis, Raffaele Palmirotta, Maurizio Volterrani, Massimo Mancone, Francesco Fedele

**Affiliations:** 1 Department of Cardiovascular, Respiratory, Nephrology, Anesthesiology and Geriatric Sciences, Sapienza University of Rome, 00161 Rome, Italy; paolo.severino@uniroma1.it (P.S.); damatoandrea92@libero.it (A.D.A.); lucrezia.netti@gmail.com (L.N.); puccimariateresa@gmail.com (M.P.); marialaurademarchis@gmail.com (M.D.M.); massimo.mancone@uniroma1.it (M.M.); 2 Department of Biomedical Sciences and Clinical Oncology Oncogenomic Research Center, ‘Aldo Moro’ University of Bari, 70124 Bari, Italy; raffaelepalmirotta@gmail.com; 3 Department of Cardiac Rehabilitation, IRCCS San Raffaele, 00163 Rome, Italy; maurizio.volterrani@sanraffaele.it

**Keywords:** ion channels, coronary blood flow, diabetes mellitus, ischemic heart disease

## Abstract

Diabetes mellitus is one the strongest risk factors for cardiovascular disease and, in particular, for ischemic heart disease (IHD). The pathophysiology of myocardial ischemia in diabetic patients is complex and not fully understood: some diabetic patients have mainly coronary stenosis obstructing blood flow to the myocardium; others present with coronary microvascular disease with an absence of plaques in the epicardial vessels. Ion channels acting in the cross-talk between the myocardial energy state and coronary blood flow may play a role in the pathophysiology of IHD in diabetic patients. In particular, some genetic variants for ATP-dependent potassium channels seem to be involved in the determinism of IHD.

## 1. Introduction

Diabetes mellitus (DM) is a complex and heterogeneous chronic metabolic disease caused by elevated levels of blood glucose. DM has a great impact worldwide: the prevalence is more than 350 million people, with 52 million in Europe. Unfortunately, those numbers refer to 2011 and will probably increase in the next decades. DM is classified into four different etiological categories: type 1, type 2, “other specific types”, and “gestational DM”. Type 1 DM (T1DM) is due to T-cell–mediated autoimmune destruction of pancreatic β-cells that leads to insulin deficiency [1]; T1DM occurs mostly in young people, generally up to 30 years of age. Type 2 DM (T2DM) is characterized by both insulin resistance and failure of pancreatic β-cells. Other specific types of DM are due to either single genetic mutations, to other pathological diseases of the pancreas, or to drugs. Gestational diabetes develops during pregnancy.

## 2. Coronary Blood Flow Regulation

From the physiological point of view, the coronary blood flow adapts itself to the metabolic and oxygen demands of the myocardium, which continuously change. The main site of coronary total resistance regulation is in the microcirculation, which is composed of small arteries and arterioles, which have diameters between 200 and 50 μm [2].

Several mechanisms regulate coronary blood flow and their contribution is different depending on the considered district. Neuro-humoral regulation and shear-stress-related vasodilatation are the main regulatory mechanisms of the epicardial arteries district, while the distal district represents the main metabolic and myogenic regulation site of coronary blood flow [2,3]. Modifying vasal tone, these mechanisms allow the coronary blood flow to adapt to cardiomyocyte metabolic demand [4].

The myocardial oxygen consumption (MVO_2_) is defined by the following formula: MVO_2_ = coronary blood flow × arterial − venous oxygen difference [5]. At rest, the myocardium has an oxygen consumption of 10 mL of oxygen, per minute, per gram of myocardial tissue and it extracts about 80% of oxygen carried by coronary blood flow [3]. Growing oxygen demand by the myocardium is satisfied with an increase of coronary blood flow through a constant modulation of coronary vascular tone. There are many important regulatory mechanisms of coronary blood flow. The autoregulation contributes to maintaining the basal vascular tone in resistance arteries, ensuring a constant blood flow to the myocardium and minimizing vascular wall stress [3]. The main action mechanism of autoregulation is therefore the myogenic regulation. From the molecular point of view, the changing of smooth muscle contraction induced by autoregulation is determined by variation in intracellular calcium values [6]. As regards the neuro-humoral regulation, the coronary arteries have both sympathetic and parasympathetic innervation. Both the innervation systems have a tonic activity, which contributes to determining the vascular basal tone. Endothelial-dependent regulation is a mechanism through which vascular resistances and vasal tone are modulated by several paracrine factors, such as nitric oxide (NO), arachidonic acid metabolic products, endothelial-derived hyperpolarizing factors (EDHF), and endothelins. Moreover, there are several hormones which contribute to coronary blood flow regulation. Among them, 17β-estradiol, progesterone, testosterone, antidiuretic hormone, and histamine determine vasodilatation [7,8,9], while growth hormone and angiotensin II are vasoconstrictors [10]. Insulin seems to have a double effect. It determines vasoconstriction, activating the sympathetic system, and also vasodilatation, inducing endothelial nitric oxide production [7]. Another important mechanism is metabolic regulation, which ensures that adequate blood flow and oxygen are provided to the myocardium in relationship with its metabolic demand [3]. This regulation mechanism has an important role in coronary vascular resistance control and it acts through several molecules produced by cardiomyocytes, such as oxygen, carbon dioxide (CO_2_), adenosine, adenine nucleotides, and reactive oxygen species, and some ion channels: the triphosphate adenosine-sensitive potassium channels (K-ATP), calcium-dependent potassium channels (KCa), and voltage-dependent potassium channels (Kv).

## 3. Role of Ion Channels in Coronary Blood Flow Regulation

Ion channels are the final effectors of the various main mechanisms involved in coronary blood flow regulation (endothelial, nervous, myogenic, and metabolic mechanisms), primarily controlled by myocardial metabolic demand [2,3]. In particular, ion channels are expressed in coronary arterial smooth muscle cells, where they directly modulate their state of contraction, and in the endothelial cells, where their importance is revealed by the secretion of vasoactive molecules such as NO. Ion channels’ primary role in coronary blood flow regulation also justifies the importance given to some specific polymorphisms in genes encoding for these proteins and their possible correlation with ischemic heart disease (IHD) [11].

Several types of channels are involved according to the type of ions. In coronary arterial smooth muscle cells, K^+^ channels determine the resting membrane potential and the cell state of contraction: when the K^+^ channels are open, the K^+^ ions go out of the cell, inducing membrane hyperpolarization and closing of Ca^2+^ channels [3]. This event causes smooth muscle cell relaxation and artery vasodilation [3]. On the other hand, closing of K^+^ channels depolarizes the membrane of the cells, activating the voltage-gated Ca^2+^ channels, inducing vasoconstriction [3]. Four types of K^+^ channels have been described in coronary vascular smooth muscle and endothelial cells: Kv, KCa, KATP, and inward-rectifier potassium (Kir) channels [2].

The critical and primary role of Kv channels is confirmed as being end effectors in the regulation of coronary blood flow at rest, during cardiac stimulation, or during catecholamine-induced raised myocardial oxygen consumption. They are also the final factors in endothelial-dependent and independent vasodilation [12,13,14,15], in which vasodilating agents make Kv channels open, through a cAMP-dependent pathway. On the other hand, vasoconstrictor agents make the Kv channels closed, allowing a Ca^2+^ ion influx. Recent evidence also underlines the Kv1.5 channel role in coronary metabolic dilation induced by mitochondrial H_2_O_2_ production [16].

KCa channels expressed in vascular smooth muscle cells (VSMCs) and endothelial cells preserve an ideal membrane potential level by controlling a normal K^+^ efflux. KCa channels can be activated both by membrane depolarization and by rises in the intracellular Ca^2+^ level [2]. In coronary artery vasculature, three types of KCa channels have been identified. They are categorized on the basis of conductance as big (B), intermediate (I), and small (S). The BKCa channels predominate mostly in VSMCs, while IKCa and SKCa do so in endothelial cells [17,18,19]. They are involved in the response of coronary smooth muscle cells to endothelial stimulation and as targets in H_2_O_2_-induced vasodilation allowed by phospholipase A2 and lipoxygenase metabolites [20,21,22,23,24]. Another important KCa channel function is to counteract vasoconstriction: in fact, several substances that mediate vasoconstriction, such as angiotensin II and endothelin, inhibit these channels [25,26,27,28]. They have a role in cardioprotection of preconditioning with ischemic injuries [29].

**Figure 1:**
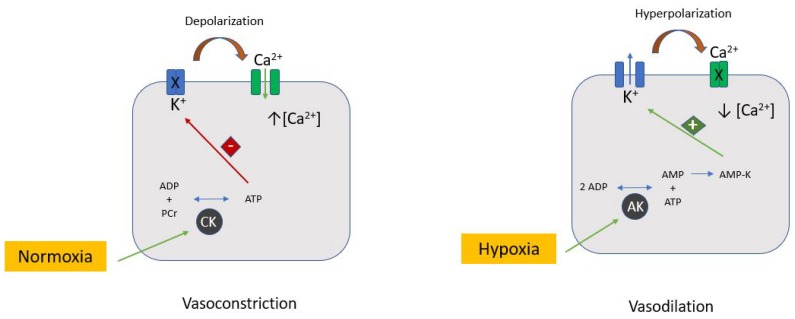
K-ATP channels and vascular tone. ATP-sensitive potassium (KATP) channels are cell membrane metabolic sensors that affect membrane excitability depending on the cellular energetic status. In normal conditions, the CK pathway is much more expressed than the AK one. A sufficient intracellular ATP concentration keeps the KATP channel closed. Hypoxia determines the activation of the AK pathway with an increase of AMP-K which opens the KATP channels. A K^+^ outflow causes hyperpolarization and, consequently, Ca^2+^ channel closing. The low intracellular Ca^2+^ concentration causes vasodilation. AK: Adenylate kinase ADP: Adenosine diphosphate; AMP-K: AMP-activated protein kinase; CK: Creatine kinase; PCr: Phosphocreatine.

KATP channels are a combination of two types of subunit: an ATP-binding cassette protein (sulfonylurea-binding subunit [SUR]) and an inward-rectifier potassium channel (Kir subunit). The Kir6.1/SUR2B and Kir6.2/SUR2A are the most represented subunit combinations forming KATP channels in coronary smooth muscle and endothelial cells, contributing to metabolism-related vascular tone modulation [2]. In fact, the reduction of intracellular ATP makes the KATP channels open and the result is an outward flux of K^+^ ions, decreasing Ca^2+^ influx and causing vasodilation. In normal metabolic conditions, KATP channels are predominantly closed. Kir subunits contribute to determining the resting membrane potential, allowing an influx of K^+^ ions faster than the outward flux. In fact, they are open in the case of membrane hyperpolarization, while a depolarized membrane potential induces their inactivation (Figure 1).

Voltage-gated sodium (Nav) channels lead to a late and peak Na^+^ current which brings cell depolarization. Coronary Nav channels are implicated in endothelial-dependent vasodilation via NO production, through the regulation of intracellular Ca^2+^ levels, and by affecting Na^+^/Ca^2+^ exchange processes [30].

Voltage-gated calcium channels (Cav) contribute to the control of coronary microvascular resistance, modulating the Ca^2+^ influx from the extracellular to the intracellular space [2], reducing coronary blood flow. Cav 1.2 is the main voltage-gated calcium channel involved in this.

**Figure 2:**
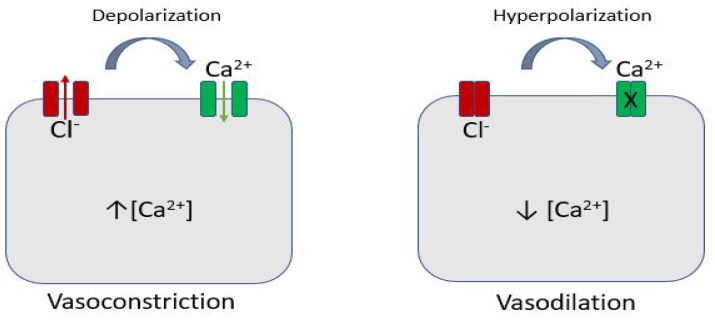
Cl^−^ channels and vascular tone. Chloride channels are expressed in the vascular smooth muscle cells: by opening these channels, Cl^−^ ions move out of the cells and, depolarizing them, determine Ca^2+^ inflow with a consequent vasoconstriction effect; the opposite occurs when they are closing. Cl^−^: Chloride.

Chloride channels expressed in the vascular smooth muscle cells can be voltage or Ca^2+^ dependent. By opening these channels, Cl^−^ ions move out of the cells and, depolarizing them, have a vasoconstriction effect; the opposite occurs when they are closing (Figure 2).

## 4. Cardiovascular Risk Factors and Ischemic Heart Disease

Among cardiovascular diseases—represented by a group of disorders of the heart and blood vessels such as IHD, cerebrovascular diseases, and peripheral arterial disease—IHD represents the most frequent cause of mortality in the world [31]. IHD consists of several clinical conditions characterized by myocardial ischemia, which is a situation of cardiomyocyte damage due to a reduced blood supply compared with their metabolic demand [31]. IHD is classically attributable to coronary artery disease (CAD), a condition characterized by the presence of an atherosclerotic plaque that causes a vascular obstruction of more than 50%. On the other hand, coronary microvascular dysfunction, which is a condition of impaired vasomotor tone due to several mechanisms, is able to provoke IHD, independently from the presence of an atherosclerotic plaque [2,11]. In fact, clinical, angiographic, and autoptic findings suggest a multifaceted pathophysiology of IHD that should not be associated only with CAD.

Several cardiovascular risk factors are involved in the pathogenesis of IHD. Diabetes mellitus is considered one of the strongest risk factors for cardiovascular disease, including IHD, cerebrovascular disease, and peripheral arterial disease [1,32,33,34,35]. Patients with DM are at double the risk of cardiovascular disease [36]. The most common cause of death among diabetic patients is cardiovascular disease, and IHD [37] is responsible for the majority of deaths. The risk of increased cardiovascular morbidity and mortality has been well described and, because of this, diabetes has been named a “cardiovascular disease equivalent”. Long-term risk of myocardial infarction in patients with diabetes was similar to that of patients with a previous myocardial infarction [38]. Moreover, one-year mortality in patients with myocardial infarction is higher in diabetic patients compared with in those without it [39]. It has been noted that patients who develop DM at a younger age present more cardiovascular complications [40]. Cardiovascular disease is the long-term complication of T1DM—as well as of T2DM—with the greatest impact on prognosis both in terms of mortality and morbidity. T1DM is associated with a markedly increased risk for IHD compared with the general population [41], similar to the increased risk associated with T2DM. Cardiovascular disease in T1DM differs from in T2DM, mostly in terms of age and gender difference. Risk factors have different influences in T1DM versus in T2DM on susceptibility to cardiovascular disease. There are several potential pathophysiological mechanisms through which diabetes causes cardiovascular disease. Usually, subjects with T2DM have other risk factors, such as hypertension as well as dyslipidemia, that are linked with increased cardiovascular risk. 

**Figure 3:**
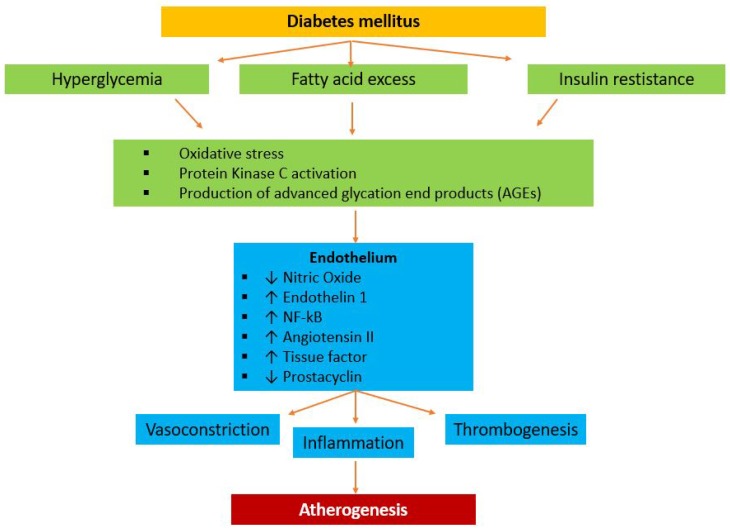
Pathophysiology of atherogenesis in patients affected by diabetes mellitus. In diabetes, the combination of hyperglycemia, free fatty acid excess, and insulin resistance leads to several systemic effects, including increasing oxidative stress, protein kinase C, and production of advanced glycation end products. The activation of these systems impairs endothelial function, through the decreasing of nitric oxide and prostacyclin and the increasing of endothelin 1, NF-κB, angiotensin II, and tissue factor. These pathways cause vasoconstriction, increase inflammation, promote thrombosis, and, thus, contribute to atherogenesis. AGEs: advanced glycation end products.

Diabetes determines a pathophysiological continuum, characterized by a state of long-standing insulin resistance with a compensatory hyperinsulinemia. Initially, the hyperglycemia remains under the threshold for the diagnosis of DM and it describes impaired glucose tolerance. Glucose metabolism impairment and endothelial dysfunction, mediated by oxidative stress and inflammation, are the main substrates of coronary atherosclerosis in diabetes mellitus [42]. A complex network of signaling pathways is involved in the multistage pathological condition leading to atherosclerosis. Imbalanced lipid metabolism and immune response lead to chronic inflammation of the arterial wall, with growth of atherosclerotic plaque [43,44]. The nature of the hyperglycemic damage in patients affected by diabetes mellitus lies in the accumulation of superoxide anions, which are free radicals capable of activating cellular pathways, included advanced glycation end products (AGEs), polyol and hexosamine flux, PKC, and vascular inflammation mediated by nuclear factor-κB. Hyperglycemia itself also increases oxidative stress through greater glucose oxidation in the citric acid cycle [45]. All these different hyperglycemia consequences lead to decreased cellular resistance to oxidative stress, amplification of the proinflammatory response, and apoptosis of endothelial cells and their overall dysfunction. These mechanisms, together with the alterations in mineral metabolism induced by renal dysfunction and the release of osteoprogenitor cells into the circulation, increase the development of vessel calcification, which is a complication of atherosclerosis in diabetic patients and correlates with increased plaque burden [46]. Diabetic patients present wide calcium deposits in coronary arteries and, thus, a bigger atherosclerotic plaque burden with a higher resulting mortality risk than in nondiabetic patients (Figure 3). Using computed tomography coronary angiography (CTCA), Van Werkhoven et al. demonstrated a higher calcium score and plaque burden in patients with DM [47]. In addition to this, studies using autopsy showed larger necrotic cores and more significant inflammation in patients with DM and acute coronary syndrome [48]. 

In diabetic patients the pathophysiology of myocardial ischemia is complex and not fully understood: some diabetic patients have coronary stenosis obstructing blood flow to the myocardium; others have coronary microvascular disease with an absence of plaques in the epicardial vessels with, or without, endothelial dysfunction. It is important to underline that myocardial ischemia is not synonymous with atherosclerotic coronary disease [49]. In the absence of coronary large vessel disease, ischemia is determined by impaired coronary vasodilator reserve and coronary microvascular disease. However, little is known about the basic aspects of diabetic coronary microvascular dysfunction. The impaired coronary arteriole vasomotion, including reduced endothelial mediated vasodilation, hypoxia-induced vasodilation, and myogenic response, are the proposed pathophysiologic processes of diabetes-induced coronary microvascular dysfunction [50]. Both hyperglycemia and insulin resistance, besides tumor necrosis factor-α overexpression and inflammation, interfere with flow-mediated endothelial-dependent vasodilation through NO level decrease and endothelin-1 level increase, which are associated with acute intracellular changes [50,51,52]. By using positron emission tomography, various studies have confirmed the reduction of endothelium-dependent and -independent vasodilator function in coronary arteries in diabetic subjects compared with a control group. These results suggest the role that chronic hyperglycemia might play in the pathogenesis of coronary microvascular dysfunction in diabetes [53]. Furthermore, an inverse correlation was shown between myocardial flow reserve and average levels of haemoglobin A1C for five years and fasting plasma glucose concentration, underlining how glycemic control is significantly related to coronary microvascular function [54]. Moreover, altered Ca^2+^ regulation with impaired myofilament function, increased reactive oxygen species formation with decreased antioxidant defenses, raised lipotoxicity, endomyocardial fibrosis, endothelial and cardiomyocyte cell necrosis and apoptosis, and autonomic dysfunction are additional mechanisms responsible for cardiomyocyte changes in diabetes mellitus [50,52,55,56]. In diabetes mellitus, chronic hyperglycemia plays a main role in the onset and progression of autonomic neuropathy, which may reduce the vasodilator effect of sympathetic stimulation on coronary resistance vessels [57].

As previously described, CAD represents the most frequent condition that leads to IHD. The presence of a coronary atherosclerotic plaque represents a pathological process that is not uniquely associated with diabetes mellitus. In fact, other cardiovascular risk factors are involved in its pathogenesis. Although they all lead to the same final condition, they act in a different way compared to diabetes mellitus, for the purposes of atherosclerosis pathogenesis. Diabetes mellitus and all the consequences of the associated hyperglycaemic condition usually combine their effects with other atherosclerotic risk factors, which add their own contribution to the pathogenesis of atherosclerosis. First, hypertension is responsible for increased vascular resistance and endothelial dysfunction in small-resistance vessels, producing a vascular remodelling [58,59]. This seems to be due to the reduction of NO release, which is caused by the abnormal production of reactive oxygen species (ROS) resultant from the cyclooxygenase (COX) activity [58,59]. The impaired NO availability has a negative impact on vasodilation, platelet adhesion and aggregation, and leukocyte migration, contributing to atherosclerotic events [60]. Besides this, atherosclerosis is closely linked to the physiological changes of aging [61]. Growing evidence confirms that cellular senescence promotes atherosclerosis [61]. In particular, senescent Endothelial Cells (ECs) show a reduction in NO production [62] and, at the same time, augmented endothelin-1 secretion [63], altered expression of the adhesion molecules VCAM-1 and ICAM-1; moreover, late-passage ECs increase the activation of nuclear factor (NF)-κB and susceptibility to apoptosis [64], leading to a shift toward a proinflammatory and proapoptotic state. These conditions contribute to and facilitate the succession of atherosclerotic phenomena [61,62,63,64]. Systemic inflammation represents a condition which is strictly associated with all the steps of atherosclerosis [65] and sometimes, such as in autoimmune disease, could be the trigger of atherosclerosis independently from other risk factors [66]. Premature atherosclerosis is demonstrated in patients with autoimmune diseases and represents the main death cause in these patients [65,66]. The dysregulation of cytokine production, mainly of IL-17, IL-6, tumor necrosis factor-α, and interferon-γ, is induced by the autoimmune response and represents the main cause of systemic inflammation and atherogenesis [65]. In patients with systemic inflammatory disorders the risk of developing atherosclerosis is also increased because the chronic inflammatory state modifies the physical features of blood, making it more viscous [66]. In these mechanisms, different molecules have a main role, such as lupus anticoagulant and anticardiolipinic antibodies in lupus erythematosus through the binding of several cell and tissue antigens [66,67]. Innate immunity seems to have a main role in the initiation of atherosclerosis through the toll-like receptor (TLR) pathways [65]. Indeed, TLR4, which is highly expressed in fragile areas of plaques, binds the oxidized low density lipoproteins (ox-LDL) supporting foam cell formation and their proinflammatory role [65]. ox-LDL stimulates the endothelial cells to produce chemokines which recruit other leucocytes around the plaque [65,68]. The inflammatory cascade at the arterial wall may be promoted by several endogenous products released by death cells such as proteins, DNA, and uric crystals with a mechanism comparable to the immune response to pathogens [65,69]. Cholesterol crystals, which are one of the main elements of atherosclerotic plaques, represent a stimulus for inflammasome activation [65]. Inflammasomes are the main effector of the innate immune response [65]. They are expressed by myeloid line cells and are made up of several proteins [65]. Inflammasomes start and support inflammation because they determine the formation of IL-1 and IL-18 and their secretion [65,70]. These cytokines support inflammation and also stimulate smooth muscle cell migration and proliferation [65,71]. In chronic inflammatory diseases, several subtypes of T lymphocytes are overexpressed and have a destructive effect on different tissues [66]. Moreover, there is a reduction of endothelial progenitor cells involved in the restoration of arterial intima [66,72]. 

## 5. Role of Ion Channels in the Pathophysiological Continuum between Cardiovascular Risk Factors and Ischemic Heart Disease

Ion channels represent the end effectors of different coronary blood flow regulatory mechanisms [3]. The effects of the different cardiovascular risk factors and genetic predisposition may cause impairing of their function and expression [2]. These conditions may be critical in the determinism of IHD [2]. In particular, altered ion channels may lead to IHD taking part in both CAD pathogenesis and in coronary microvascular dysfunction [2,11].

As evidenced by international literature, the impairment of several ion channels has been associated with predisposition to diabetes mellitus. A study by Su et al. showed that AGEs could be able to cause vascular damage both in a direct and an indirect way [73]. As regards the former, AGEs determine the impairing of Kv channel activity in vascular smooth muscle cells through glycation reaction. As regards the latter, the interaction between AGEs and their receptors represents a stimulus for oxidative stress, which is associated with a downregulation of Kv 1.2 and Kv 1.5 proteins and mRNA of expression [74,75,76]. These mechanisms cause the alteration of coronary blood flow regulation through the impairing of both non-endothelial- and endothelial-dependent vasodilatation mechanisms [73].

Moreover, KCa channel activity has been described as compromised both in T2DM and in T1DM [77]. A study by Li et al. showed that the molecular base of impaired KCa channel activity is the higher BK-β1 subunit ubiquitination [78]. However, nuclear factor E2-related factor-2 (Nrf2) regulates the redox state of cells and its expression is reduced because of oxidative stress associated with T2DM [78]. The lower expression of Nrf2 is associated with a lower inhibition of muscle ring finger protein 1 (Murf1) activity [78]. Given that Murf1 is an E-3 ligase involved in ubiquitination mechanisms, it determines the increase of BK-β1 ubiquitination and the resulting KCa channel impairment [78]. Liu et al. added in vitro NS309—a SKCa/IKCa activator—to diabetic patient arterioles, observing lower endothelial-dependent vasodilatation compared with arterioles from nondiabetic patients [79]. However, they demonstrated a normal expression and localization of these types of coronary microvasculature endothelial channels in diabetic patients, underlining that T2DM might cause a downregulation of these channels through post-translational modifications [79]. Moreover, in mice with T2DM, coronary BKCa potassium current dysfunction has been associated with higher and more extensive myocardial ischemia/reperfusion damage [80].

Regarding K-ATP channels, they are also strongly expressed on α and β pancreatic islet cells, where they show Kir6.2 and SUR1 subunits. The former subunit is encoded by the *KCNJ11* gene and the latter by the *ABCC8* gene [81]. Pancreatic K-ATP channels modulate insulin and glucagon pancreatic islet production, playing a main role in the regulation of glucose homeostasis [82]. K-ATP channels are normally open and determine an outward potassium current which keeps the β cell in a hyperpolarized state. When the glycemic levels increase, the glucose is carried inside the β cell by the GLUT2 transporter. The increase in glucose β cell concentration stimulates ATP production through aerobic glycolysis and other metabolic pathways. The increasing levels of intracellular ATP cause the inhibition of K-ATP channels, a reduction in outward potassium current, and the consequent β cell depolarization. The depolarization determines voltage-gated calcium channel activation, which causes an increase in intracellular calcium levels and the following insulin granules exocytose [82]. K-ATP channels seem to also have a main role in the simultaneous regulation of α cell activity. As regards α cells, K-ATP closure is paradoxically related to cell hyperpolarization and glucagon secretion inhibition [82,83]. K-ATP channel function is strictly associated with insulin and glucagon secretion and, therefore, with blood glucose levels. Several examples demonstrate how mutations and single-nucleotide polymorphisms (SNPs) of K-ATP subunit encoding genes may cause a dysregulation of channel activity, determining a higher or lower risk of developing type 2 diabetes mellitus—and, therefore, cardiovascular disease—in humans. The polymorphism Glu23Lys of KCNJ11 together with Ser1369Ala of *ABCC8* gene, with which it is in linkage disequilibrium, relate to a higher risk of developing T2DM [84,85]. The Glu23Lys polymorphism may predispose T2DM through reduced glucagon secretion suppression induced by glucose [86]. KCNJ11 and ABCC8 gain-of-function mutations relate to a rare type of diabetes mellitus, which arises before the first year of life: neonatal diabetes mellitus [82]. In this syndrome, the increased K-ATP channel function determines reduced β cell depolarization, which causes lower insulin secretion in response to the increase of glucose blood levels [87,88,89]. The EK and KK genotypes of the E23K allele, also named rs5219, of KCNJ11 reduce β cell function and represent an independent risk factor for the onset of the prediabetes in youth [83], a condition defined by the presence of lower blood glucose fasting levels than the diabetes mellitus threshold, but higher than normal ones [90,91]. Furthermore, the rs5219 polymorphism only predisposes young females to T2DM [83]. A study by Rastegari et al. underlined the association between the KK homozygous genotype of the E23K polymorphism of the *KCNJ11* gene and T2DM in adults [92]. Qiu et al. confirmed that the rs5219 polymorphism of KCNJ11 predisposes to T2DM, mostly in Caucasians [93]. On the other hand, Souza et al. demonstrated that the same polymorphism E23K (rs5219) was not associated with type 1 or 2 diabetes mellitus in Euro-Brazilian subjects [94]. A meta-analysis performed by Qin, L.J. et al. of KCNJ11 and ABCC8 polymorphisms revealed that rs5219 (KCNJ11) represents the most strictly associated SNP with T2DM in the global population [95]. The same study also revealed a correlation among rs5215 and rs5210 SNPs of the *KCNJ11* gene and the rs757110 SNP of the *ABCC8* gene and predisposition to developing T2DM [95]. Emdin et al. confirmed the results of previous studies about the role of ABCC8 p.A1369S missense mutation in reducing the risk of developing T2DM despite it causing predisposition to a high body mass index (BMI) [96]. The presence of the S allele in the population plays the same role as antidiabetic therapy with sulfonylurea [97]. Emdin et al. demonstrated that the presence of the S allele of ABCC8 p.A1369S is also associated with a lower risk of developing coronary artery disease despite the elevated BMI. The effect determined by the S allele on T2DM and cardiovascular risk is due to a more diffuse body fat distribution and to glucose homeostasis improvement [96]. 

In coronary circulation, impaired ion channel function has been associated with alteration of endothelial-dependent and -independent vasodilation [12,13,14,15]. In the section of coronary vasculature in which the endothelium is dysfunctional, there is impaired NO activity [98], a higher expression of inflammatory markers, and an increased tendency to the onset of atherosclerotic lesions [99,100,101,102,103,104,105]. The endothelial dysfunction is associated both with higher expression of vasoconstrictor factors, such as endothelin-1 and angiotensin II, and with lower expression of vasodilator factors, such as NO and endothelial-derived repolarizing factor (EDRF) [106]. The increase of coronary vascular tone is linked to the alteration of coronary blood flow physical features, a condition which promotes several mechanisms which are at the base of atherosclerosis pathogenesis such as platelet, lymphocyte, and macrophage aggregation and inflammation [107,108,109,110,111,112,113].

The KCa 3.1 channel is expressed by VSMCs and it regulates their cell cycle and proliferation [113,114]. In conditions such as DM, this channel is upregulated and stimulates VSMC proliferation and migration [113,115], causing the onset and growth of atherosclerotic lesions [113,116,117]. The same channel KCa 3.1 is expressed also by macrophages, lymphocytes, and platelets [113]. Its activation is associated with the durable increase of intracellular calcium levels, which stimulates cell recruitment and proliferation, cytokine secretion, and, therefore, inflammatory cascade amplification [118]. At the arterial wall, all these biological events promote the initiation and progression of atherosclerotic lesions [113]. The KCa3.1 channel is not expressed in monocytes but is highly expressed in macrophages, which constitute the atherosclerotic plaque [118]. The same channel has a main role in M1 macrophage polarization and increases atherosclerotic plaque instability [118]. 

The Kir2.1 channel is expressed by macrophages and, by hyperpolarizing the membrane potential, it determines the increase of intracellular calcium levels through the activation of the alfa 1H T-type calcium channel [119,120]. The inward calcium current may stimulate the expression of several proteins, among which the scavenger receptor [120]. Therefore, the Kir2.1 channel may promote ox-LDL intake by macrophages and foam cell formation, regulating scavenger receptor expression [120].

The Kv1.3 channel is expressed both by vascular smooth muscle cells, where it stimulates their proliferation and migration [121,122], and by lymphocytes, where it promotes proliferation and cytokine production [122,123,124,125]. Kan et al. studied the role of the Kv1.3 channel in macrophages and in the pathogenesis of atherosclerosis [122]. They showed high expression of the Kv1.3 channel in macrophages of patients with acute coronary syndrome, hypothesizing a role of this channel in the initiation and progression of atherosclerosis and in the instability of the plaques [122]. The upregulation of Kv1.3 channel expression and function may stimulate greater activation of extracellular signal-regulated kinase (ERK) through its phosphorylation [122]. ERK belongs to the mitogen-activated protein kinase (MAPK) family and mainly promotes macrophage recruitment into the arterial intima [122]. 

**Figure 4:**
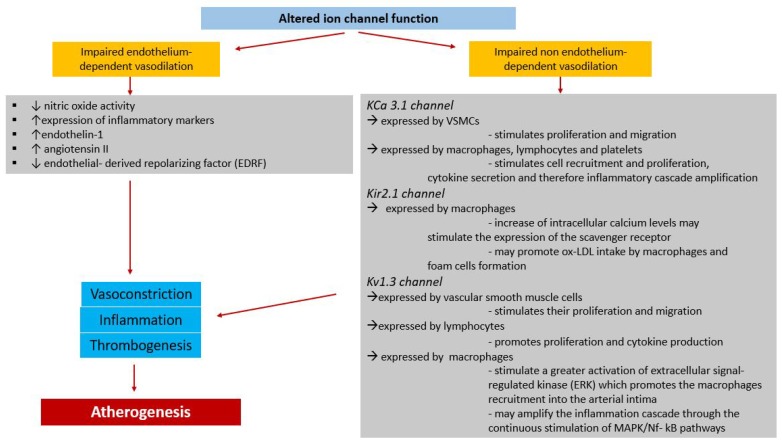
Contribution of altered ion channel function to the pathophysiology of atherogenesis. In coronary circulation, ion channels contribute to both endothelium-dependent and non-endothelium-dependent vasodilation. When ion channel function is impaired, the vasodilating effect is decreased, affecting both pathways. In the left side of the figure (grey box), molecules and proteins involved in the impaired endothelium-dependent vasodilation are listed. These pathways are responsible of vasoconstriction, inflammation, and thrombogenesis. On the right (grey box), there is a focus on the main K channels involved in the amplification of the inflammatory cascade through the augmented proliferation and migration of lymphocytes, macrophages, and platelets. All these mechanisms contribute to atherogenesis.

Ling et al. demonstrated that the upregulation of K-ATP channels expressed by macrophages has a main role in the instability and vulnerability of atherosclerotic plaques [126]. Indeed, it may amplify the inflammation cascade through the continuous stimulation of MAPK/NF-κB pathways [126] (Figure 4).

We reported elsewhere [11] a gain-of-function mutation in KATP channels that is negatively associated with IHD in Caucasians. In particular, we described the possible correlation of single-nucleotide polymorphisms (SNP) in genes encoding for some coronary blood flow effectors (i.e., ion channels as KATP, Kv, Nav, endothelial nitric oxide synthase, and Sarcoplasmic/endoplasmic reticulum calcium ATPase [SERCA] pump) with susceptibility to IHD. Comparing patients with coronary artery disease (CAD), microvascular dysfunction, and normal coronary arteries [11], the SNPs rs5215_GG, rs5218_CT, and rs5219_AA for the gene *KCNJ11* encoding for Kir6.2 subunit of KATP were more frequently observed in patients with anatomically and functionally normal coronary arteries. On the other hand, the rs1805124_GG genotype of *SCN5A* gene encoding for the Nav1.5 channel was more frequently observed in patients with CAD; finally, no association between the studied SNPs of SERCA pump, Kir6.1 subunit of KATP, and the Kv1.5 channel and the presence of IHD was observed [11]. On the other hand, no difference in terms of the presence of diabetes mellitus was observed between patients with CAD, microvascular dysfunction, and normal coronary arteries, giving more significance to the genetic differences observed between the groups.

Regarding the SNPs for KCNJ11, rs5219_AA was associated with the presence of normal coronary arteries, as a protective independent factor for microvascular dysfunction with a statistical trend [11]. In addition, the SNP rs5215_GG was associated with the presence of normal coronary arteries [11] and showed a significant potential protective role in the determinism of coronary artery disease. We performed a post hoc analysis of data from our previous study comparing subgroups of patients with and without DM in correlation with the genetic profile of the studied ion channels. In Table 1, the basic clinical data are shown. 

Regarding genetic aspects, in the post hoc analysis we observed that rs5215_GG was more frequently observed in patients without T2DM compared with haplotypes G/A (*p* = 0.0001) and A/A (*p* = 0.0001), whereas rs5219 did not show any statistically significant correlation with diabetes mellitus. Moreover, in nondiabetic patients, rs5215_GG was more frequently associated with the presence of normal coronary arteries, compared with CAD (*p* = 0.0001) or with microvascular dysfunction (*p* = 0.0001). On the other hand, the presence of an A allele in position 1009 of KCNJ11 (haplotypes G/A or A/A) was associated with the presence of T2DM (*p* = 0.0001) and, in particular, in patients with CAD compared with microvascular dysfunction (*p* = 0.0001) as well as with normal coronary arteries (*p* = 0.0001). As is well known, K-ATP channels are expressed in several different districts, including pancreas and coronary circulation, where they are involved in many physiological processes that may, or may not, be linked to a unique pathophysiological continuum. At the moment, there is no evidence on the functional association between the presence of SNPs for K-ATP channels, T2DM, and IHD. In fact, taking into consideration the central function of K-ATP channels in the regulation of coronary vascular tone and in pancreatic functions, the observed correlations between some genetic variants and clinical phenotypes need to be confirmed by electrophysiology analysis, such as the patch clamp method, since basic investigations that examine SNPs and K-ATP channel function are not available so far.

## 6. Conclusions

Regulation of coronary blood flow is heterogeneous and complex. Several ion channels play a pivotal role in this regulation and in its homeostasis. Alteration in these mechanisms can lead to IHD, which represents one the most frequent cause of mortality in the world [31]. By convention, IHD is the expression of CAD; however, clinical, angiographic, and autoptic findings show that coronary microvascular dysfunction, impairing vasomotor tone, is able to provoke IHD independently from the presence of an atherosclerotic stenosis. In the determinism of IHD, diabetes mellitus is one of the strongest risk factors. However, the pathophysiology of myocardial ischemia in diabetic patients is not fully understood. In the last decades, an increasing number of studies have shown the importance of ion channels as end effectors of several regulation mechanisms, such as coronary blood flow and glucose homeostasis. Impaired ion channel activity might be associated with the alteration of these regulatory mechanisms, causing predisposition to several pathologies. In this work, we mainly focused on the pathophysiological role of SNPs of coronary artery and pancreas islet cell ion channels in the determinism of both diabetes mellitus and IHD. Through the systematic review of the most recent international literature, we highlighted that there are some ion channels whose impaired function is strongly associated with diabetes mellitus and IHD susceptibility (Table 2). We also highlighted the most important SNPs that modify these channels’ activity, with particular reference to the rs5219 and rs5215 polymorphisms of KCNJ11 which we also studied in our previous work [11]. We previously identified [11] the SNP rs5215_GG in the gene *KCNJ11*, encoding for Kir6.2 subunit of KATP channels, as a protective independent factor for CAD in Caucasians. Performing a post hoc analysis on subgroups of diabetic and nondiabetic patients, diabetes mellitus was not significantly associated with either acute coronary syndromes (i.e., ST elevation myocardial infarction, non-ST elevation myocardial infarction, unstable angina), presence of CAD, or normal coronary arteries. However, nondiabetic patients more frequently showed microvascular dysfunction. These observed clinical associations are in contrast with those classically described as associated with diabetes mellitus, which is one of the strongest risk factors for ACS, CAD, and microvascular dysfunction.

Regarding genetic aspects, the SNP rs5215_GG for the Kir6.2 subunit of KATP channels was statistically associated with the absence of DM. Moreover, in our population, SNP rs5219 did not present any significant correlation with diabetes mellitus, different to observations made by some authors [83,92,93,95]. Interestingly, in nondiabetic patients, the presence of rs5215_GG was more frequently observed in patients with normal coronary arteries, although they present other cardiovascular risk factors such as hypertension and dyslipidemia, compared with patients with CAD and with patients with microvascular disease. Taking in mind that our previously enrolled population [11] was at high cardiovascular risk with an indication to undergo coronary angiography for suspected myocardial ischemia, if we consider only nondiabetic patients, the presence of normal coronary arteries seems to depend mostly on the presence of a particular genetic variant of KCNJ11 rather than other cardiovascular risk. In particular, we observed that the presence of an A allele in position 1009 of KCNJ11 was associated with the onset of T2DM and IHD due to CAD, while the SNP rs5215_GG is more frequently observed in nondiabetic patients and normal coronary artery patients. On the other hand, we previously reported [11] that rs5215_GG represents an independent protective factor for the development of CAD, both in diabetic and nondiabetic patients.

These observations suggest that genetic variants of KATP channels seem to have a role in the pathophysiology of IHD and of DM. However, functional data on the pathophysiological link between the presence of these SNPs for K-ATP channels, DM, and IHD are not available so far. Moreover, these observations should be confirmed on a greater population, including analysis of other genes encoding for molecular mechanisms involved in the regulation of coronary blood flow.

In conclusion, evidence from the literature and from our observations suggests that some SNPs for K-ATP channels might influence the presence of DM as well, as they seem to be involved in the coronary blood flow regulation that, in turn, influences susceptibility to IHD. Unfortunately, there is not a univocal and clear explanation for it, so far. It is probably that several other molecular factors act in this pathophysiological continuum. Our observations are only a small piece in a big puzzle.

## Figures and Tables

**Table 1:** Baseline data of groups.

Items	DM (Tot. 67)	NDM (Tot. 175)	*p* Value
Gender [male] % (*n*)	68.6 (46)	64.5 (113)	NS
Smoke habit % (*n*)	34.3 (23)	41.1 (72)	NS
Hypertension % (*n*)	86.5 (58)	63.4 (111)	0.0002
Dyslipidemia % (*n*)	88 (59)	64 (112)	0.0002
Family history of CVD % (*n*)	80.5 (54)	70.2 (123)	NS
ACS % (*n*)	58.2 (39)	51.4 (90)	NS
CAD % (*n*)	70.1 (47)	61.7 (108)	NS
CMD % (*n*)	8.9 (6)	22.8 (40)	0.016
NORM % (*n*)	20.8 (14)	15.3 (27)	NS

DM: diabetic patients; NDM: nondiabetic patients. NS: nonsignificant results (*p* > 0.05). CVD: cardiovascular disease. ACS: acute coronary syndrome (i.e., ST elevation myocardial infarction, non-ST elevation myocardial infarction, unstable angina). CAD: coronary artery disease (i.e., epicardial stenosis >50% at angiography). CMD: coronary microvascular dysfunction (i.e., reduced coronary flow reserve at functional tests by intracoronary acetylcholine and adenosine infusion). NORM: anatomically and functionally normal coronary arteries.

**Table 2:** List of single-nucleotide polymorphisms of genes encoding for different ion channels and their correlation with ischemic heart disease.

Ion Channel	Protein	Gene	SNPs	Effect	References
K-ATP	Kir6.2	*KCNJ11*	rs5210	- Predisposition to develop T2DM	[95]
K-ATP	Kir6.2	*KCNJ11*	rs5215_GG	- Association with anatomically and functionally normal coronary arteries - Significant potential protective role in the determinism of coronary artery disease in Caucasianss - Statistical association with the absence of DM	[11]
K-ATP	Kir6.2	*KCNJ11*	rs5218_CT	- Association with anatomically and functionally normal coronary arteries	[11]
K-ATP	Kir6.2	*KCNJ11*	rs5219_AA; rs5219 (E23K-Glu23Lys)	- Association with anatomically and functionally normal coronary arteries - Statistically protective independent factor for microvascular dysfunction - Reduction of β cell function and independent risk factor for the onset of the prediabetes in youth - Different Authors do not agree about the presence or the absence of any significant correlation with diabetes mellitus - Predisposition to T2DM through reduced glucagon secretion suppression induced by glucose - Linkage disequilibrium with the polymorphism Ser1369Ala of *ABCC8* gene	[11,84,85,86,92,93,94]
K-ATP	SUR1	*ABCC8*	rs757110	- Predisposition to develop T2DM	[95]
K-ATP	SUR1	*ABCC8*	p.A1369S	- Reduction of the risk to develop T2DM and coronary artery disease despite it causing predisposition to a high BMI - The effect determined by the S allele on T2DM and cardiovascular risk is due to a more diffuse body fat distribution and to glucose homeostasis improvement	[96,97]
K-ATP	SUR1	*ABCC8*	Ser1369Ala	- Association with a higher risk to develop T2DM, as component of KATP channel in regulation of insulin secretion from pancreatic β cell membranes	[84,85]
Nav	Nav1.5	*SCN5A*	rs1805124_GG	- More frequently observed in patients with CAD	[11]

K-ATP: ATP-sensitive potassium channels. Nav: Voltage-gated sodium channel. SNPs: single-nucleotide polymorphisms. T2DM: Type 2 diabetes mellitus. BMI: body mass index. CAD: coronary artery disease.

## Abbreviations

DM	diabetes mellitus
T1DM	type 1 diabetes mellitus
T2DM	type 2 diabetes mellitus
MVO_2_	myocardial oxygen consumption
NO	nitric oxide
K-ATP	triphosphate adenosine-sensitive potassium channels
KCa	calcium-dependent potassium channels
Kv	voltage dependent potassium channels
Kir	inward-rectifier potassium channel
Nav	voltage-gated sodium channels
Cav	voltage-gated calcium channels
VSMCs	vascular smooth muscle cells
AGEs	advanced glycation products
ECs	endothelial cells
ox-LDL	oxidized LDL
ROSIHD	reactive oxygen speciesischemic heart disease
CAD	coronary heart disease
SNPs	single-nucleotide polymorphisms
